# A robust, cost-effective and widely applicable whole-genome sequencing protocol for capripoxviruses

**DOI:** 10.1016/j.jviromet.2022.114464

**Published:** 2022-03

**Authors:** Elisabeth Mathijs, Andy Haegeman, Kris De Clercq, Steven Van Borm, Frank Vandenbussche

**Affiliations:** Infectious Diseases in Animals, Sciensano, Rue Juliette Wytsmanstraat 14, 1050, Brussels, Belgium

**Keywords:** Capripoxvirus, Whole-genome sequencing (WGS), Long-range PCR, Next-generation sequencing (NGS), Nanopore sequencing, Single-Molecule real-time sequencing (SMRT)

## Abstract

The diseases caused by capripoxviruses (CaPVs) are of major economic concern in sheep, goat and cattle as they are inexorably spreading into non-endemic regions. As CaPV strains are serologically indistinguishable and genetically highly homologous, typing closely related strains can only be achieved by whole genome sequencing. Unfortunately the number of publicly available genomes remains low as most sequencing methods rely on virus isolation. Therefore, we developed a robust, cost-effective and widely applicable method that allows to generate (nearly) complete CaPV genomes directly from clinical samples or commercial vaccine batches. A set of pan-CaPVs long-range PCRs spanning the entire genome was designed to generate PCR amplicons that can be sequenced on commonly used high-throughput sequencing platforms: MiSeq (Illumina), RSII (PacBio) and MinION (Oxford Nanopore Technologies). The robustness of the LR-PCR strategy was evaluated for all 3 members of *CaPV* directly from a variety of samples, including clinical samples (N = 7), vaccine batches (N = 6), and virus isolates (N = 2). The sequencing method described here allows to reconstruct (nearly) complete CaPV genomes in less than a week and will aid researchers studying closely-related CaPV strains worldwide.

## Highlights

• Robust method for the genomic characterization of all *Capripoxvirus*es.
• Pre-sequencing enrichment method based on targeted long-range PCR amplification.
• Method applicable to low titre samples such as blood samples and vaccine batches.
• Viral DNA enrichment compatible with various sample types and sequencing platforms.
• Complete coding genome sequencing evaluated on three different sequencing platforms.

## 1 Introduction

Capripoxviruses (CaPV) represent one of the nine genera currently recognized within the *Chordopoxvirinae* subfamily of the *Poxviridae* family. The genus *Capripoxvirus* is comprised of lumpy skin disease virus (LSDV), goatpox virus (GTPV), and sheeppox virus (SPPV). All CaPVs are responsible for economically important diseases in domestic ruminants that threaten the livelihoods of smallholder farmers and poor rural communities in endemic regions (Tuppurainen et al., 2017a; Tuppurainen and Oura, 2012) and recently also farming communities in free regions (Tuppurainen et al., 2017b). Due to their potential for rapid spread and substantial economic impact, the World Organization for Animal Health (OIE) has categorized all CaPV diseases as notifiable.

Sheeppox (SPP) and goatpox (GTP) are endemic in small ruminants in Central and North Africa, the Middle East, and various parts of Asia. In cattle, lumpy skin disease (LSD) was initially confined to the African continent and the Middle East but recently spread to Europe and Asia (Roche et al., 2020; Tuppurainen et al., 2017a). All three diseases have made several incursions in southern Europe but were successfully controlled by stamping out or mass vaccination. In 2019, LSD reached the Eastern part of Asia being reported in India, Bangladesh and China (Gupta et al., 2020; Lu et al., 2020) and in 2020 the disease spread further to South-East Asia (Flannery et al., 2021; Roche et al., 2020; Tran et al., 2021). The recent spread of LSDV into disease-free areas in the Eurasian continent has brought renewed attention to these neglected diseases.

The CaPV genome consists of a linear double-stranded DNA molecule that is about 150 kb in length and encodes between 147 and 156 open reading frames (ORFs). The nucleotide composition is approximately 75 % A + T and uniformly distributed along the entire genome. The genome structure is similar to that of other chordopoxviruses with the central region containing the more conserved genes that are involved in basic viral replicative processes. By contrast, genes that are species- or host-specific are distributed toward the end of the genome (Gubser et al., 2004). Two identical inverted terminal repeats (ITRs) are located at both ends of the genome. *Capripoxvirus* genomes are very similar to each other with a sequence identity of no less than 96 % across their entire length (Tulman et al., 2002, 2001).

As the different CaPV species cannot be distinguished morphologically or serologically, CaPVs were originally classified according to the host species from which they were isolated. Although most SPPV or GTPV isolates cause more severe disease in either sheep or goats, some isolates are equally pathogenic in both animal species (Babiuk et al., 2008). Moreover, some wild ruminants are known to be susceptible to LSDV (Greth et al., 1992; Le Goff et al., 2009; Young et al., 1970), which further complicates this host-based classification system. Reliable differentiation between the three CaPV species is therefore only possible at the molecular level. Several real-time PCR (qPCR) assays have been developed to detect and differentiate CaPV species. All of these assays are based on the detection of species-specific single nucleotide polymorphisms (SNPs) but target different regions within the genome, namely the G-protein-coupled chemokine receptor (GPCR) gene (Lamien et al., 2011), the 30 kDa RNA polymerase subunit (RPO30) gene (Gelaye et al., 2013) or the homolog of the variola virus B22R gene (Chibssa et al., 2019). Although these qPCR assays allow to identify the different CaPV species, they lack the discriminatory power to distinguish closely related strains (Mathijs et al., 2016c). Differentiation at strain level requires a much higher resolution that can only be obtained by comparing whole genome sequence data.

Unfortunately, CaPVs are difficult to sequence due to their large genome size, skewed nucleotide composition, and the presence of multiple homopolymeric and repetitive sequences. Although clinical samples may contain high viral loads, viral DNA represents only a small fraction of the total DNA that is present in these samples. To increase the signal (viral genome) to noise (host genome) ratio, most CaPV sequencing protocols require high concentrations and relatively large volumes of viral DNA that can be obtained only by virus isolation. Similar to other poxviruses, the first CaPV genomes were obtained using a shotgun sequencing approach. Viral DNA was isolated from infected cell cultures and randomly fragmented by incomplete enzymatic digestion. DNA fragments within a predefined size range were cloned and sequenced using Sanger’s dideoxy chain termination method and a primer walking strategy (Kara et al., 2003; Tulman et al., 2002, 2001). With the advent of high-throughput sequencing, the labor-intensive cloning step could be omitted which greatly reduced the cost and time needed to obtain complete genomes. To increase the number of viral reads, the viral DNA is enriched before sequencing by purifying and/or concentrating the virus particles (Biswas et al., 2020; Douglass et al., 2019; Flannery et al., 2021; van Schalkwyk et al., 2020; Wolff et al., 2020; Zeng et al., 2014). Although these protocols allow to measure genetic variation on a genome-wide scale, they still rely on virus isolation to obtain sufficient viral-enriched DNA. Unfortunately, virus isolation is time consuming and requires appropriate cell-culture and biocontainment facilities that are not available to all laboratories.

This study aimed to develop a robust, cost-effective and widely applicable method that allows to generate (nearly) complete CaPV genomes directly from clinical samples or commercial vaccine batches. Taking advantage of the genetic similarity of CaPVs, a set of pan-CaPVs long-range PCRs (LR-PCRs) was developed that spans the entire genome using only a limited number of amplicons. The resulting PCR amplicons can be sequenced on all currently available high-throughput sequencing platforms to reconstruct (nearly) complete genomes in less than a week.

## 2 Materials and methods

### 2.1 Samples

All three CaPV species and different sample matrices (cell culture, vaccine batch, and clinical sample) were included in this study (Table 1). The attenuated LSDV strain SA-Neethling was kindly provided by Dr. E. Tuppurainen (at that time working at The Pirbright Institute, United Kingdom). The isolate was passaged three times in OA3.Ts cells (ATCC: CRL-6546) grown in Dulbecco's Modified Eagle's Medium (Life Technologies) supplemented with 5% foetal calf serum (Life Technologies), 0.10 mg/l streptomycin, and 100 000 IU/l penicillin. Other samples consisted of a skin lesion from an experimentally infected cow with the attenuated LSDV strain SA-Neethling (bioethical approval # 20150605−01), as well as cell culture or clinical samples from recent LSDV field strains and CaPV vaccine batches from different manufacturers (Table 1).

**Table 1:** Overview of the *Capripoxvirus* complete coding sequences determined using the methodology developed in this study. The sequencing strategy (PCR enrichment and sequencing technology) is clarified for each strain.

ID	Strain	Species	Sample type	Amplicon length	Sequencing platform	GenBank accession (SRA Bioproject)	Reference
SA-Neethling	Attenuated LSDV strain SA-Neethling	LSDV	Cell culture	5500−7500	MiSeq (7500) RSII (7500) MinION (7500)	MW435866 (PRJNA661421)	This study
		LSDV	Skin sample	7500	–	–	
Caprivac	Gorgan	GTPV	Vaccine batch	5500−7500	RSII (5500)	KX576657	(Mathijs et al., 2016a)
Jovivac	Yugoslavian RM65	SPPV	Vaccine batch	5500−7500	–	KJ818284 KJ818291	(Tuppurainen et al., 2014)
Kenyavac	KSGP 0240	LSDV	Vaccine batch	5500	RSII	KX683219	(Vandenbussche et al., 2016)
Lumpyvax	Attenuated strain SIS	LSDV	Vaccine batch	5500	RSII	KX764643	(Mathijs et al., 2017, 2016b)
Herbivac	Attenuated strains SA-Neethling					KX764644	
LSD OBP						KX764645	
Evros/GRC/15	Field strain Greece 2015	LSDV	Cell culture	7500	MiSeq	KY829023	(Agianniotaki et al., 2017)
Kubash/KAZ/16	Field strain Kazakhstan 2016	LSDV	Skin sample	7500	MiSeq	MN642592 (PRJNA587601)	(Mathijs et al., 2020b)
219−249/BUL/16	Field strain Bulgaria 2016	LSDV	Blood sample	7500	MiSeq	MT643825 (PRJNA641001)	(Mathijs et al., 2020a)
20L42_Quyet-Thang/VNM/20	Field strain Vietnam 2020	LSDV	Skin sample	7500	MiSeq	MZ577073	(Submitted for publication)
20L43_Ly-Quoc/VNM/20						MZ577074	
20L70_Dinh-To/VNM/20						MZ577075	
20L81_Bang-Thanh/VNM/20						MZ577076	

LSD(V) = lumpy skin disease (virus); GTPV = Goatpox virus; SPPV = Sheeppox virus.

OBP = Onderstepoort Biological Products.

PacBio = Pacific Biosciences; ONT = Oxford Nanopore Technologies.

### 2.2 DNA purification

Sample treatments prior to DNA extraction differed according to sample type. Twenty-five milligrams of skin sample were homogenized in 180 μL phosphate-buffered saline (PBS) and 25 μL Puregene proteinase K using a TissueLyser (Qiagen) followed by an overnight incubation at 56 °C at 200 rpm on a ThermoMixer C (Eppendorf). The freeze-dried vaccine pellets were simply resuspended in 3 mL PBS and used immediately.

To eliminate host DNA, the skin homogenate, PBS dissolved vaccine pellets, and cell culture supernatant were treated with Baseline-ZERO DNase (Epicenter) according to the manufacturer’s instructions. The remaining DNA was extracted using a Puregene extraction kit (Qiagen) according to instructions provided by the manufacturer. The viral load of the purified DNA samples was assessed using a probe-based qPCR assay designed to detect all CaPVs in the conserved D5R region (Haegeman et al., 2013).

### 2.3 Primer design for long-range PCR amplification

Nine full-length genome sequences representing all 3 CaPV species (Supplementary Table 1) were downloaded from GenBank and aligned using MAFFT v7.305b (Katoh and Standley, 2013). At first, the alignment was used to design 30 primer pairs that generate tiled amplicons of about 5.0–5.5 kbp in size (Supplementary Table 2). At a later stage, the number of primer pairs was reduced so that the entire genome could be covered by only 23 overlapping amplicons of about 7.5 kbp in size (Table 2).

**Table 2:** Primer sequences for long-range PCR amplification and expected amplicon sizes for species compromising the *Capripoxvirus* genus.

F	Primer name	Primer sequence (5′-3′)	Expected size (bp)^a^		
			GTPV	LSDV	SPPV
1	fp CaPV-F1	AAACCTGTAAATGGATACTTTTTTCATTC	7339−459	7566−68	7403−11
	rp CaPV-F1	ATTTGGAAATATAATTGTGTTAACTGTTCT			
2	fp CaPV-F2	TGTGAAAAATTAATCCATTCTTCTAAACAG	7584−9	7652−62	7594−617
	rp CaPV-F2	TACATACATTTCAAGTACTAAAGAGAAGGAA			
3	fp CaPV-F3	ATGTCAACAACATTTTTGCTATTCAATG	7639−53	7713−20	7575−6
	rp CaPV-F3	TTTTGGCCAGATATTTACAATGCTATCA			
4	fp CaPV-F4	GATGGACCTAATGGAGTTATTATTGAG	7622	7621−2	7620−3
	rp CaPV-F4	GAAAAATCAAATGTAAACAAACAGCTGT			
5	fp CaPV-F5	ATTCCATCATTGTTTGGTATTATTCCA	7617−8	7608−11	7597
	rp CaPV-F5	ACATATCATGTAAATAATAATAACGGAACAAC			
6	fp CaPV-F6	GCTGAAGAATATGAATACAATACGCTAT	7618−22	7619−20	7631−2
	rp CaPV-F6	AAAAAACAAAATTTGAAGAACCTAAATCAG			
7	fp CaPV-F7	GTATTGTTCTCCAAGTTTTACATCCTT	7557−61	7556−59	7570−1
	rp CaPV-F7	CACATTTCTATTTTTAATAAATACGATTCCTTTC			
8	fp CaPV-F8	ATCTCCGTTTGCTAAAAAAGATAAAGC	7667−76	7675	7690−1
	rp CaPV-F8	CATCTATGATAAATCGCACTATGGGTTTTA			
9	fp CaPV-F9	TGGGACCCAAATTGTTCAGAATCTAA	7682−97	7688−95	7701−2
	rp CaPV-F9	TTTCTAACAATGGCCAAAAACGTTTATAA			
10	fp CaPV-F10	ATCCCACTTAAGATAATAAGATTTTTTAGAAAC	7549−52	7549−50	7555−6
	rp CaPV-F10	TTCCTCAGATTATCCGCTAATTTATTTGA			
11	fp CaPV-F11	CTCTCTAATTTTAGTTATGTTTTCATCTATCCA	7621−39	7593−9	7603−9
	rp CaPV-F11	AGAAAGGAATATTATATGCCCTATAGATATAGA			
12	fp CaPV-F12	CCCAGATAAAGACGCAATAAGTAGAT	7668−71	7668	7669
	rp CaPV-F12	AACGAGTTGTTAGTCATTTGAGATAC			
13	fp CaPV-F13	ACCATTCCAGAATTAAGTTTGATAATAAAC	7698	7700	7703
	rp CaPV-F13	GGGGGAAATTATTTCAGAGTTATTAGAT			
14	fp CaPV-F14	TATTATCATCGTTTCCAATTAATGAATTAATTAC	7547	7559−608	7550−6
	rp CaPV-F14	CAACGAAAAACATTGATAAAATCTGATG			
15	fp CaPV-F15	CTTTGATGTTGTTACCACCTTTCC	7771−2	7792	7773
	rp CaPV-F15	TTAAAAGAACAAAGTGGAATGGTAAGATAG			
16	fp CaPV-F16	GAGTAAGATTTGATTTTTGAGATGCTTG	7627−8	7638−45	7622
	rp CaPV-F16	TTTTCAAACCCGTATTCATTTTTTACTG			
17	fp CaPV-F17	ACTAAGGTTTGTTTTAGATAAATGGGAT	7515−6	7510−5	7517−643
	rp CaPV-F17	GTTTACAATTCTGCATATGATAGTTATATATGG			
18	fp CaPV-F18	GCCATCTAACTCTATTGTTAAATCCA	7553−65	7571−99	7518−20
	rp CaPV-F18	CCTTTGCCTAAATCATCATTTTTCTC			
19	fp CaPV-F19a	CCRTCTATAGATATTAGAATTGTTAGTAAACC	7567−81	7711−5	7626−7
	fp CaPV-F19b	CCGTCTATAGATATTAGAATTATTAGTAAACC			
	rp CaPV-F19	GTATACATGATATTAGTGCAACATTGTTATG			
20	fp CaPV-F20	GTTTGTATGATGCCAGATTCAGATATTAC	7610−77	7623−7	7576−604
	rp CaPV-F20	TAAACATAGACTCTTCTTTCGGTAGAC			
21	fp CaPV-F21	GTTCGGTTTCATATTTTTAGCATATTCAC	7601−68	7650−707	7612−39
	rp CaPV-F21	TGATATAAGTTTCATCCAAAAATCATATGTTG			
22	fp CaPV-F22	GATTTACMCCACTTTTATCTTCTGTATATG	7771−85	7782−852	7738−67
	rp CaPV-F22	GACATATTAGATTTTGGAAATATAAGAGGTG			
23	fp CaPV-F23	CCAAAAACGATTTCATTGTATAAAGAAC	7375−9	7417−22	7279−87
	rp CaPV-F23	AAACCTGTAAATGGATACTTTTTTCATTCAATC			

F, fragment; fp, forward primer; rp, reverse primer; CaPV, capripoxvirus; GTPV, goatpox virus; LSDV, lumpy skin disease virus; SPPV, sheeppox virus.

a expected amplicon sizes based on 9 full genome sequences of genus *Capripoxvirus* (GPV n = 3, LSDV n = 3, SPV n = 3) available at http://www.viprbrc.org/.

### 2.4 Long-range PCR amplification

LR-PCR reactions were set up as follows (all mentioned concentrations are final concentrations): 1x Q5 High-Fidelity DNA Polymerase reaction buffer (New England Biolabs), 1 M betaine, 0.5 μM of both forward (pf) and reverse (pr) primer, 0.4 mM CleanAmp dNTPs (TriLink Biotechnologies), 1 U of Q5 High-Fidelity DNA polymerase, 5 μL of DNA template, and distilled water up to a volume of 50 μL. Cycling conditions were as follows: 98 °C for 3 min (initial denaturation), 35 cycles of 10 s at 98 °C, 30 s at 63 °C and 7 min at 72 °C (amplification), followed by 2 min at 72 °C (final elongation). The PCR products were stained with Midori Green Direct (Nippon Genetics) before being visualized on 1% agarose gels. Only single products of the expected size were purified using the Agencourt AMPure XP system (Beckman Coulter) according to the manufacturer’s instructions. Purified DNA was quantified on a Quantus Fluorometer (Promega) using the QuantiFluor dsDNA System (Promega).

### 2.5 Library preparation and sequencing

**Fig. 1:**
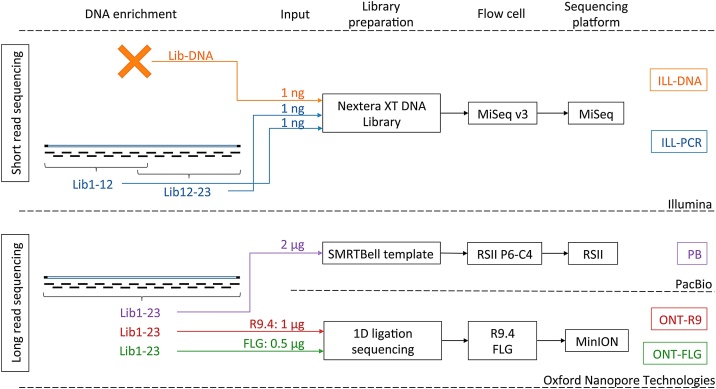
**Schematic overview of the different sequencing strategies applied to the attenuated LSDV strain SA-Neethling in the study.** ILL-DNA (orange) and ILL-PCR (blue) refer to Nextera XT Illumina sequencing of a library prepared from extracted DNA without any enrichment or from pools of 12 amplicons corresponding to half of the *Capripox* genome, respectively. PB (purple) refers to PacBio sequencing of a library prepared from a pool of 23 amplicons. ONT-R9 and ONT-FLG refer to MinION (Oxford Nanopore Technologies) sequencing of a library prepared from a pool of 23 amplicons on a R9.4 Flow Cell or a Flongle Flow Cell, respectively.

To assess the robustness and wide applicability of the protocol, 3 sequencing platforms relying on different technologies were evaluated in the present study (Fig. 1).

#### 2.5.1 Illumina (ILL) sequencing

In order to distinguish the short sequence reads from both ITRs, two libraries (Lib1−12 and Lib12−23), each comprising a pool of 12 LR-PCR amplicons corresponding to half of the CaPV genome, were prepared using a Nextera XT DNA Library Preparation Kit (Illumina) according to the manufacturer’s instructions. An additional library (Lib-DNA) was prepared directly from 1 ng of purified DNA (i.e. without PCR enrichment). The libraries were quantified with a Kapa Library Quantification Kit (Roche) and the insertion size was verified on a Bioanalyzer 2100 system using a High-Sensitivity DNA Kit (Agilent Technologies). Twelve femtomoles of each barcoded library were pooled prior to sequencing on a MiSeq System using the MiSeq Reagent Kit version 3, 2 × 300 bp (Illumina). Sequencing was performed at the Neuromics Support Facility -VIB Genomics Core (UAntwerp, Belgium).

#### 2.5.2 PacBio (PB) sequencing

The LR-PCR amplicons covering the entire CaPV genome were pooled in equimolar quantities to a final amount of 2 μg for single-molecule real-time (SMRT) sequencing (PacBio). SMRTbell libraries were prepared using the SMRTbell Template Prep Kit 1.0 (PacBio). To filter out smaller fragments and SMRTbell dimers, the libraries were size selected on a BluePippin 0.75 % Gel cassette (Sage Science) using the "High Pass V3″ protocol (collection protocol settings: 5.5 kb – 9.5 kb). P6-C4 sequencing was performed using a single SMRT cell on a PacBio RSII sequencer with a movie time of 240 min (PacBio) at the Genomics Core Leuven (KU Leuven, Belgium).

#### 2.5.3 Oxford Nanopore Technologies (ONT) sequencing

Nanopore sequencing libraries were prepared with a SQK-LSK109 1D Ligation Sequencing Kit (Oxford Nanopore Technologies) according to the protocol provided by the manufacturer, using either 500 ng (Flongle Flow Cell) or 1000 ng (R9.4.1 Flow Cell) of equimolarly pooled amplicons. The resulting libraries were bead purified with AMPure XP beads (Beckman Coulter), quantified using the QuantiFluor® dsDNA System (Promega) and 20 and 120 fmol were loaded onto separate Flongle or R9.4.1 Flow Cells, respectively (Oxford Nanopore Technologies). Sequencing runs were performed using MinKNOW software v3.5.5 (without real-time base calling) for 24 h.

### 2.6 Sequence data analysis

#### 2.6.1 Illumina sequence data

The raw MiSeq sequence data from each library, representing either half of the genome (PCR amplicon pools, Lib1−12 and Lib12−23) or the entire genome (purified DNA, Lib-DNA), were analysed separately and referred to as ILL-PCR and ILL-DNA, respectively. The quality of the raw data was assessed using FastQC v0.11.3 (http://www.bioinformatics.babraham.ac.uk/projects/). Trimming was performed using Trim Galore v0.6.6 (http://www.bioinformatics.babraham.ac.uk/projects/trim_galore/) based on quality (Q score >30) and length (length >80 bp, 5′ clip for R1 and R2 = 30 bp). To obtain near full-length genomes, the trimmed reads were *de novo* assembled using SPAdes v3.13.0 with optimized k values and a subsample of 10,000–20,000 paired-end reads (Bankevich et al., 2012). For Lib1−12 and Lib12−23, the consensus sequences from both libraries were merged into a single contig using Cap3 version date 02/10/2015 (Huang and Madan, 1999).

#### 2.6.2 PacBio RSII sequence data

For the PacBio RSII data analysis, quality filtering was performed using SMRT Portal v2.3.0 (PacBio) with the following settings: minimum subread length = 3000, minimum polymerase read quality = 80, and minimum polymerase read length = 3000. Consensus sequences for each PCR amplicon were obtained *de novo* using the “RS_Long_Amplicon_Analysis.1″ protocol included in SMRT Portal v2.3.0. with default parameters except for the Maximum Number Of Subreads which was set to 4000.The resulting amplicon sequences were assembled into a single contig using iAssembler software v1.3.2 (Zheng et al., 2011).

#### 2.6.3 Nanopore sequence data

HAC base calling of the raw ONT reads was performed with Guppy software v3.4.3 (ONT). Quality assessment and filtering of the ONT reads were performed using NanoPlot v1.27.0 and NanoFilt v2.5.0, respectively (De Coster et al., 2018). Quality filtering parameters were set as follows: minimum length = 6000 bp; maximum length = 8000 bp; quality = 12; headcrop = 50 bp. The reads were assembled *de novo* using canu v2.1 with the following parameters: genomeSize = 150k, readSamplingCoverage = 100, contigFilter = “2 0 1.0 0.5 0″, and correctedErrorRate = 0.105 (Koren et al., 2017). Polishing of the longest consensus sequence was performed with medaka v0.8.0 (https://github.com/nanoporetech/medaka) using all quality filtered/trimmed reads.

#### 2.6.4 Comparison of assembled sequence data

The quality of the *de novo* assemblies obtained from the different sequencing strategies was assessed and compared using Quast v5.0.2 (Mikheenko et al., 2018). Coverage depth across the SA-Neethling genome was plotted in R using the ggplot2 package (v4.0.2) (Wickham, 2016).

### 2.7 Genome finishing and annotation

#### 2.7.1 Sequencing of genome termini and Sanger sequencing confirmation

Like all poxviruses, CaPV genomes contain a stretch of nucleotides, called functional resolution sequence (FRS), that is situated between the telomeres and the first tandem repeat (Merchlinsky, 1990). This highly conserved sequence was used to amplify and sequence the 5′ and 3′ termini of the CaPV genome with primer pair: FRS TTTTATAGGCTTAAAAAAAAGTATAATATTG and ORF1 ATTTTAGCAAGAGCAGCAGAATATTGG. The PCR reaction was set up as follows (final concentrations): 1x Q5 High-Fidelity DNA Polymerase reaction buffer (New England Biolabs), 1 M betaine, 0.5 μM of both FRS and ORF1 primers, 0.4 mM CleanAmp dNTPs (TriLink Biotechnologies), 1 U of Q5 High-Fidelity DNA polymerase, 5 μL of DNA template, and distilled water up to a volume of 50 μL. Cycling conditions were as follows: 98 °C for 3 min (initial denaturation), 35 cycles of 10 s at 98 °C, 30 s at 63 °C and 1 min at 72 °C (amplification), followed by 2 min at 72 °C (final elongation).

Discrepancies between the full-length consensus sequences and previously published vaccine-associated Neethling genomes (n = 7; accession numbers: AF409138, MN636838−43) were confirmed or refuted by dideoxy chain terminator sequencing (Sanger sequencing). Sequencing was performed on an Applied Biosystems 3130 Genetic Analyzer Sequencer using the BrightDye Terminator Cycle Sequencing kit (Nimagen) according to the manufacturer’s instructions.

#### 2.7.2 Genome annotation

Open reading frames were predicted using both NCBI ORF-Finder (http://www.ncbi.nlm.nih.gov/orffinder/) and GATU relative to the Neethling vaccine LW 1959 sequence (AF409138). The annotated full genome sequence can be accessed at GenBank accession number MW435866.

## 3 Results

### 3.1 Primer design and long-range PCR amplification

**Fig. 2:**
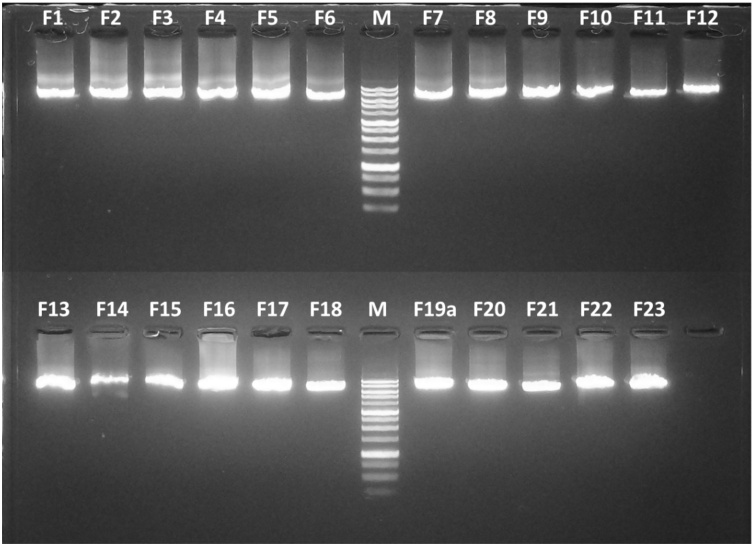
1% agarose gel electrophoreses analysis of the 23 long range PCR amplicons for the attenuated LSDV strain SA-Neethling cell culture isolate. M = molecular weight marker range 250 – 10,000 bp.

Due to the AT-rich nature of the CaPV genome, finding suitable regions for primer design was challenging. Nevertheless, taking advantage of the high genetic relatedness between CaPV species, we were able to select primer sets sharing similar annealing temperatures (63 °C) and generating similar amplicon lengths (5.5 kbp) (Table 2, Supplementary Table 2). The LR-PCR protocol was optimized to obtain only single amplicons of the expected size that overlap each other by at least 1 kb and cover the entire coding CaPV genome (Mathijs et al., 2016a, 2016b; Vandenbussche et al., 2016). Crossing point (Cp) values obtained from extracted DNA in the CaPV-D5R qPCR assay ranged between 13.43 and 29.52. To further reduce costs and hands-on-time, the PCR protocol was modified by increasing the amplicon lengths from 5.5 kbp to 7.5 kbp, which greatly reduced the number of amplicons needed to cover the entire genome. Twenty-three single amplicons of 7.5 kbp were obtained for each of the CaPV DNAs and different sample types (LSDV attenuated SA-Neethling isolate, Fig. 2; GTPV Caprivac vaccine batch, Supplementary Fig. 1; SPPV Jovivac vaccine batch, Supplementary Fig. 2; LSDV attenuated SA-Neethling skin sample, Supplementary Fig. 3). DNA from LSDV field strains Evros/GR/15, Kubash/KAZ/16, 210LSD-249/BUL/16, 20L42_Quyet-Thang/VNM/20, 20L43_Ly-Quoc/VNM/20, 20L70_Dinh-To/VNM/20, 20L81_Bang-Thanh/VNM/20 was amplified similarly (Agianniotaki et al., 2017; Mathijs et al., 2020a, , submitted for publication). In total, successful *Capripoxvirus* genome PCR enrichment was demonstrated for six skin lesion samples, one blood sample, two cell culture supernatants, and six vaccine batches (Table 1). For all CaPV samples, the minimum required amount of purified DNA per amplicon (250 ng) was obtained allowing the preparation of equimolar amplicon pools for both short (MiSeq-Illumina) and long-read sequencing (RSII-PacBio, MinION-ONT) platforms. The coding sequences of the different vaccine batches and clinical samples from Greece, Kazakhstan, and Bulgaria were already reported previously (Agianniotaki et al., 2017; Mathijs et al., 2020b,). The sequencing results from the pooled 7.5 kbp amplicons and extracted DNA of the attenuated LSDV SA-Neethling isolate were further detailed in this study to evaluate the impact of the different sequencing strategies.

### 3.2 Data output from the different sequencing strategies

The data output obtained from the different sequencing strategies for the attenuated LSDV SA-Neethling isolate after quality filtering/trimming is summarized in Table 3. MiSeq sequencing of the Nextera XT (Illumina) libraries Lib-DNA, Lib1−12, and Lib12−23 generated reproducible read numbers and read lengths. The raw sequencing data for these libraries have been submitted to the Short Read Archive under the BioProject number PRJNA661421. As reported earlier for MiSeq Reagent Kit version 3 (2 × 300 bp), the quality of the second read (R2) was systematically lower than that of the first read (R1), which resulted in shorter reads after quality trimming. The data generated by the SMRTcell loaded with a single SMRTbell (PacBio) library is in line with expectations with over 70,000 reads with an average length of 14 kb. The R9.4.1 MinION Flow Cell (Oxford Nanopore Technologies) generated 1,241,611 base-called reads from which 579,266 were left after quality trimming/filtering. Due to the poor quality of the majority of the reads generated, only 5173 out of the 78,844 Flongle reads remained after quality filtering (Table 3).

**Table 3:** Sequencing data obtained for the cell-cultured attenuated SA-Neethling LSDV strain with the different sequencing strategies applied in this study.

Output Stats	Short-read data: Illumina^1^			Long-read data: PacBio^2^ - ONT^3^		
	Lib-DNA	Lib1−12	Lib12−23	PacBio	ONT-R9.4	ONT-FLG
# bases	120,756,296	159,153,635	171,532,167	1,002,374,879	4,342,829,196	38,787,394
	73,537,623	101,446,577	113,149,263	956,205,996		
# reads	566,987	701,789	749,787	71,593	579,266	5173
	566,987	701,789	749,787	138,929		
Av read length	212.98	226.78	228.77	14,001	7497	7498
	129.7	144.55	150.91	6882		
N50 read length	222	250	246	26,269	7543	7542
	134	154	160	7752		

1 Paired-end data is given R1 and R2, respectively. Read trimming parameters (Trim_Galore!): Quality = 30; Length = 80; clip_R1 and clip_R2 = 20.

2 Read and subread stats are given. Read filtering parameters (“RS_Subreads.1″ protocol in SMRT Portal): Minimum Polymerase Read Length and Mininum Subread Length = 3000 bp; Minimum Polymerase Read Quality = 0.8.

3 Read filtering parameters (NanoFilt): length = 6000 bp; maxlength = 8000 bp; quality = 12; headcrop = 50 bp.

As expected, over 99.9 % of the reads obtained after PCR enrichment (ILL-PCR, PB and ONT) map to the CaPV genome whereas only 9.3 % of the ILL-DNA (DNA from a virus isolate without enrichment) reads correspond to CaPV. The majority of the ILL-DNA reads were of ovine origin, reflecting the cell line used for virus propagation (data not shown).

### 3.3 Read assembly, assembly quality assessment, and genome coverage

To assess the advantages and limitations of the different sequencing platforms, the resulting *de novo* assemblies were evaluated for accuracy and completeness using QUAST (Table 4, Gurevich et al., 2013). Mismatches and insertions/deletions (InDels) of the *de novo* assemblies were determined relative to the reference sequence MN636842 (van Schalkwyk et al., 2020). Amplicon sequencing data from the different sequencing platforms (ILL-PCR, PB, ONT-R9.4.1, ONT-FLG) could be assembled into a single, near full-length consensus sequence whereas the *de novo* assembly of data generated without PCR enrichment (ILL-DNA) only yielded a multitude of contigs (Table 4). Nevertheless, the ILL-DNA dataset allowed to cover 95.86 % of the mapped reference genome. The most accurate consensus sequences were obtained for the ILL-PCR and PB assemblies, both in terms of number of mismatches and number of InDels per 100 kb. Due to the low basecalling accuracy in homopolymer regions, both the ONT-R9.4.1 and ONT-FLG assemblies contained a markedly higher number of InDels with no less than 110.45 and 56.56 indels per 100 kb, respectively.

**Table 4:** *De novo* assembly statistics obtained from the data generated by the different sequencing strategies used to determine the coding genome of SA-Neethling. Statistics are based on contigs of size > = 5000 bp, unless otherwise noted (e.g., "# contigs (> = 0 bp)" include all contigs).

Assembly stats	ILL-DNA	ILL-PCR	PB	ONT-R9.4	ONT-FLG
# contigs (> = 0 bp)	23,405	1	1	1	1
# contigs	14	1	1	1	1
Largest contig	35,563	150,242	150,280	150,382	150,231
Reference* length	150,396	150,396	150,396	150,396	150,396
GC (%)	29.76	25.91	25.91	25.90	25.92
Reference* GC (%)	25.91	25.91	25.91	25.91	25.91
Genome fraction (%)	95.86	99.91	99.93	99.93	99.93
# mismatches* per 100 kbp	24.97	21.96	22.62	27.28	25.28
# indels* per 100 kbp	11.79	11.31	11.31	110.45	56.56

ILL = Illumina, PB = PacBio, ONT = Oxford Nanopore Technologies.

* Reference : Lumpy skin disease virus isolate LSD-220−2-NW-RSA-1993 (MN636842).

**Fig. 3:**
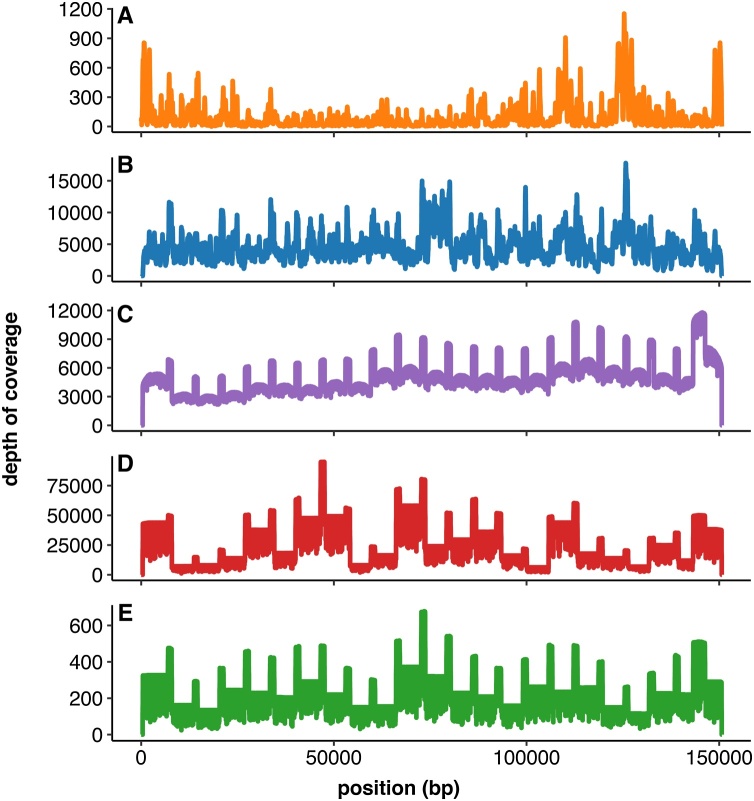
**Depth of coverage of the attenuated LSDV strain SA-Neethling genome for all **sequencing platforms that were used in this study.**** (A) Nextera XT Illumina sequencing of a library prepared from extracted DNA without any enrichment (ILL-DNA). (B) Nextera XT Illumina sequencing of a library prepared from 2 pools of 12 amplicons each corresponding to half of the Capripox genome (ILL-PCR). (C) PacBio sequencing of a library prepared from a pool of 23 amplicons (PB). (D) MinION sequencing of a library prepared from a pool of 23 amplicons on a R9.4 Flow Cell (ONT-R9). (E) MinION sequencing of a library prepared from a pool of 23 amplicons on a Flongle Flow Cell (ONT-FLG).

Fig. 3 depicts the depth of coverage of the SA-Neethling genome for all sequencing platforms that were used in this study. The coverage depths were highly variable between platforms but mainly rely on the sequencing effort per sample. For ILL-DNA and ILL-PCR, the average depth of coverage was 95.5 and 4676, respectively. For ILL-DNA, 24.6 % of the bases were covered by less than 20 reads whereas the minimum coverage for ILL-PCR was 67. High variation in coverage depth across the genome was observed for ILL-DNA and ILL-PCR due to the enzymatic tagmentation step during Nextera XT library preparation (Fig. 3 A, B). As there is no fragmentation required for sequencing on third-generation platforms (PB and ONT), all 23 amplicons and their overlaps can be clearly identified on the coverage plots (Fig. 3 C, D, E).

### 3.4 Genome finishing, genome annotation and comparison with published Neethling strain genomes

Genome finishing was performed on the consensus sequence that was generated from the ILL-PCR and PB contigs. The genome termini and discrepancies with closely related Neethling strains were analysed by Sanger sequencing. When aligned to publicly available full-length LSDV genomes, the SA-Neethling consensus sequence shared over 99.9 % nucleotide identity with LSDVs in subgroup 1.1, which contains both Neethling-type vaccine viruses and virulent vaccine-associated viruses from South Africa (van Schalkwyk et al., 2020). Only 53 variable sites (23 SNPs and 29 InDels) were observed between the SA-Neethling consensus sequence and the Neethling vaccine LW1959 (AF409138). All variable sites and their impact on ORF lengths or amino acid changes are given in Supplementary Table 3. Ninety-one percent (n = 21) of the SNPs were located in coding regions of the genome with 76 % of them (n = 16) resulting in an amino acid change. Similarly, most of the InDels (82 %, n = 24) were located in one of the ORFs. Compared to the Neethling vaccine LW1959, nine InDels caused a frameshift that disrupted the stop codon and resulted in elongation of the ORFs LW019, LW026, LW086, LW087, LW131, LW134, and LW144. Two internal amino acids were inserted in ORF LW022, while 3 amino acids were deleted in ORF LW076.

## 4 Discussion and conclusions

In contrast to many RNA viruses, CaPVs display only low levels of genetic diversity both within and between species. Although sequencing specific regions (e.g. RPO30 and GPCR) allows to discriminate vaccine from field strains, none of these regions contain sufficient information to study possible lines of transmission within an outbreak or epidemic (Mafirakureva et al., 2017; Molini et al., 2018; Sudhakar et al., 2020). Comparison of the whole genome sequences of recent LSDV isolates from the 2015–2016 epidemic in southern Europe revealed only a limited number of point mutations between the isolates (Agianniotaki et al., 2017; Mathijs et al., 2020b,). Moreover, the characterization of full-length LSDV genomes isolated from the field exhibit probable recombination events between wild-type and vaccine LSDV isolates (Sprygin et al., 2020, 2018). Whole genome sequencing is therefore essential when comparing closely related CaPV strains in outbreak or epidemic investigations and the identification of recombinant CaPVs as it allows to capture all genetic variation at once.

The advent of HTS technologies has enabled researchers to measure genetic variation on a genome-wide scale at single nucleotide resolution (Mathijs et al., 2016c). Theoretically, these novel technologies are ideal to study large DNA viruses such as CaPVs. Unfortunately, the proportion of viral nucleic acids in clinical samples is usually very small compared to the host-derived nucleic acids. As a consequence, direct sequencing of all nucleic acids in a sample (i.e. metagenomics) rarely generates sufficient viral reads to reconstruct the entire genome or study the diversity within the viral population. The results obtained with the unenriched sample (Lib-DNA) are therefore not surprising. Although we were able to cover 95 % of the genome, the depth of coverage was highly variable across the genome, ranging from 1 to 1152 reads (Fig. 3A). However, it should be noted that a high titre cell cultured viral stock was used in the present study. Less coverage will be expected for clinical samples or vaccine batches (low titre cell culture). A viral enrichment step is therefore indispensable if one wishes to distinguish variable sites reliably. Over the years, several enrichment strategies have been developed to focus sequencing efforts on the viral nucleic acids of interest. These include (i) virus isolation and concentration, (ii) host nucleic acid depletion, (iii) target capture using virus-specific probes and (iv) virus genome amplification using conserved primer sets.

We specifically chose a genome amplification strategy as it is a well-established method that is relatively straightforward and both highly specific and sensitive. In contrast to other CaPV sequencing methods that are based on virus isolation and concentration, our genome amplification strategy does not require specialized equipment or technical skills and can be easily performed in any laboratory that is equipped with a thermocycler. Thanks to the high genetic similarity of CaPVs, we were able to design a set of pan-CaPV LR-PCRs that cover nearly the entire genome using only 23 amplicons. To demonstrate the robustness of our method, we successfully tested it on SPPV, GTPV and LSDV isolates, clinical samples and vaccine batches. A similar tiling amplicon approach has been used previously for the amplification of several other viral genomes (Freed et al., 2020; Gardner et al., 2014; Grubaugh et al., 2019; Li et al., 2012; Quick et al., 2017; Yu et al., 2020) and has also been developed by the ARTIC network to sequence the severe acute respiratory syndrome coronavirus 2 (SARS-CoV-2). Although this strategy is in theory applicable to any virus species, most tiling amplicon protocols target relatively small RNA viruses such as Ebola virus, Zika virus or West Nile virus. As far as we know, no tiling amplicon sequencing protocols have been described for large DNA viruses such as CaPVs. Due to the size (150 kb) and complex structure (repetitive regions) of CaPV genomes, we opted for an amplicon size of 7.5 kb instead of the much smaller sizes that are generally used in tiling amplicon protocols. As explained by Quick et al. (2017), the likelihood of finding large genome fragments in a sample is determined mainly by the type of virus (RNA/DNA virus, high/low viral load, etc.). Fortunately, CaPV virions are relatively stable in the environment and can be found at very high concentrations in clinical samples such as skin lesions. Routine analyses in our laboratory have shown that the LR-PCR strategy is very robust and can be applied to a wide variety of sample matrices including isolates, clinical samples and vaccine batches. By increasing the amplicon size, we were able to reduce the number of amplicons to 23 and, consequently, also decrease the cost and time needed to amplify an entire CaPV genome. Moreover, the larger amplicon sizes allow to cover the entire ITR in a single amplicon, which facilitates downstream data analysis. In contrast to most tiling amplicon protocols that use only 2 primer pools, we decided to amplify each region individually to facilitate the identification of amplicon dropouts and, at the same time, assess the yield of each reaction individually prior to sequencing. Although this singleplex approach is more expensive, it guarantees a more uniform coverage across the entire genome that cannot be achieved using highly multiplexed tiling PCRs. For instance, several studies have described highly uneven genome coverage when using the ARTIC SARS-CoV-2 amplification protocol (Cotten et al., 2021; Itokawa et al., 2020; Lam et al., 2021; Tyson et al., 2020).

As amplicon-based enrichment strategies provide both enrichment and amplification at the same time, our CaPV LR-PCR can be easily coupled to any of the currently available sequencing platforms. Although a detailed comparison between the different sequencing platforms is beyond the scope of this study, the observed results are in line with previous studies. The most accurate consensus sequences were obtained for the ILL-PCR and PB assemblies, which yielded identical sequences. Despite its markedly lower raw read accuracy, the uniform distribution of errors across PB reads allows to perform a consensus correction step using all available long reads. Unfortunately, PB platforms are expensive and not always easily accessible due to scarcity of service providers. As reported previously, both ONT assemblies suffered from numerous InDel errors that are a well-known problem of nanopore sequencing (Van der Verren et al., 2020). It was shown that nearly half of the errors are due to homopolymers, especially those longer than 4 (Delahaye and Nicolas, 2021). These errors also explain the noise observed in the ONT coverage plots. Although the N50 read length of the ONT data was approximately 7500 bp, large drops in coverage depth were observed across the entire length of the amplicons due to the inaccurate calling of homopolymer stretches. Due to these limitations, despite being the most accessible sequencing technology, ONT nanopore sequencing is presently less suitable for the accurate characterization of CaPV genomes. However, as the sequencing chemistry and the data analysis are evolving continuously, the accuracy of nanopore sequencing is expected to improve over the following years. For instance, new flow cells, sequencing kits, basecalling algorithms and polishing tools are being developed to tackle these InDel artefacts, particularly in homopolymer regions. Additional experiments will be needed to assess the impact of the continuous developments in nanopore sequencing and data analysis.

In conclusion, we have developed a robust, cost-effective and widely applicable method to sequence complete CaPV coding genomes. The described LR-PCR strategy allows to enrich viral target DNA of any CaPV species directly from a variety of samples, including clinical samples (tissue lesions, blood), vaccine batches, and virus isolates. The enriched DNA can be sequenced on all currently available sequencing platforms without additional amplification, yielding (nearly) complete genomes in less than a week. As over 99 % of the DNA is CaPV-specific, only low sequencing efforts are needed. As a consequence, high numbers of CaPV samples can be multiplexed on a single sequencing run, which greatly reduces the sequencing cost per sample. In our experience, 50−100 K paired 250 bp reads on a MiSeq run are sufficient for complete genome sequencing, which represents only a fraction of the sequencing effort that is needed to cover the entire CaPV genome without amplicon-based enrichment. We hope that the method described in this study will aid researchers worldwide that need to study closely-related CaPVs (e.g. outbreak investigations).

## Data availability

We have reviewed published papers, most of which have not yet made their raw EEG data available.

Data will be made available on request.

## Author_statement

Elisabeth Mathijs: Conceptualization, Methodology, Software, Formal analysis, Writing – Original/Draft

Andy Haegeman: Resources

Kris De Clercq: Supervision, Funding acquisition

Steven Van Borm: Writing – Review & Editing

Frank Vandenbussche: Conceptualization, Methodology, Software, Formal analysis, Writing – Original/Draft

## Declaration of Competing Interest

The authors declare that they have no known competing financial interests or personal relationships that could have appeared to influence the work reported in this paper.
