# Supplementary material for: A robust, cost-effective and widely applicable whole-genome sequencing protocol for capripoxviruses
Source: J Virol Methods. 2022 Mar;301:114464. doi: 10.1016/j.jviromet.2022.114464 (PMC8872832; doi:10.1016/j.jviromet.2022.114464)
Supplement: Supplementary file 1 [file mmc1.docx]

Supplementary Table 1. *Capripoxvirus* genomes used for the design of the primer pairs for the long-range PCRs.

| Capripoxvirus | Isolate name | GenBank Accession number |
| --- | --- | --- |
| LSDV | Neethling 2490 | NC_003027 |
|  | Neethling Warmbaths LW | AF409137 |
|  | Neethling vaccine LW 1959 | AF409138 |
| SPPV | 10700-99 strain TU-V02127 | NC_004002 |
|  | A | AY077833 |
|  | NISKHI | AY077834 |
| GTPV | Pellor | NC_004003 |
|  | G20-LKV | AY077836 |
|  | FZ | KC951854 |
