# Supplementary material for: A robust, cost-effective and widely applicable whole-genome sequencing protocol for capripoxviruses
Source: J Virol Methods. 2022 Mar;301:114464. doi: 10.1016/j.jviromet.2022.114464 (PMC8872832; doi:10.1016/j.jviromet.2022.114464)
Supplement: Supplementary file 2 [file mmc2.docx]

Supplementary table 2. Primer sequences for long-range PCR amplification resulting in 30 overlapping amplicons of circa 5.5 kb covering the entire Capripoxvirus coding sequence.

| F | Primer name | Primer sequence |
| --- | --- | --- |
| 1 | pf_CaPV-F1 | GATACTTTTTTCATTCAATCTTTTAAGTC |
|  | pr_CaPV-F1 | TCACCCACTAGCCAAGGA |
| 2 | pf_CaPV-F2 | ATGTATCAACTTTCCTAGGCG |
|  | pr_CaPV-F2 | CATTTATTTTCTATTTACTGATTATCAATGTT |
| 3 | pf_CaPV-F3 | TTTACTTCATAAGAACAATTAAACGTAT |
|  | pr_CaPV-F3 | GTTGATGAAGTAGATCTTTCTAAGG |
| 4 | pf_CaPV-F4 | TATCCATTTTCTAGCCCATTCAG |
|  | pr_CaPV-F4 | CCTATAATCGATGATGAATTAGAAAC |
| 5 | pf_CaPV-F5 | TTTTCATAATGCTCTAGTAATTCGTTAT |
|  | pr_CaPV-F5 | CGACAAGATACCATGCAATCA |
| 6 | pf_CaPV-F6 | CACTAGATAATGAACTTTCATCTAAATAA |
|  | pr_CaPV-F6 | ATATCCTTTTTTAGCGTCTACTCT |
| 7 | pf_CaPV-F7 | ATTAAATCAAATGATATATCTGTAACACTA |
|  | pr_CaPV-F7 | CATGTAAATAATAATAACGGAACAACA |
| 8 | pf_CaPV-F8 | TATCATTACCAACTGATTTGTCTACT |
|  | pr_CaPV-F8 | ATTGTTGATGTAGATGGTAAAAGTG |
| 9 | pf_CaPV-F9 | AAAACTCCAAATATTGCAGAATACA |
|  | pr_CaPV-F9 | CTTTGTTCCCGCATAGATTTAG |
| 10 | pf_CaPV-F10 | CTCATCTGGGATAGTTTTTGTTTC |
|  | pr_CaPV-F10 | GAACTTTAACACTTATGGCTCC |
| 11 | pf_CaPV-F11 | ACACAGATAGAGCCTCAAAG |
|  | pr_CaPV-F11 | GAGTTATCATCATTAGTTAGATCGAA |
| 12 | pf_CaPV-F12 | AAAGTTTAAATAACAAGATTGTGGTAG |
|  | pr_CaPV-F12 | TCAGTAGCATTAATTTCTTCCTGA |
| 13 | pf_CaPV-F13 | GATTTAAACACCTACATCTTATATCG |
|  | pr_CaPV-F13 | CCAATTACGTGAAACTTTTCATAATA |
| 14 | pf_CaPV-F14 | ATGGCAGACGATAATCTAATTAC |
|  | pr_CaPV-F14 | GGTTGTATAAATACTAATTCTACTTCATG |
| 15 | pf_CaPV-F15 | GCGTTAAATAAAGGAATTGATTATGA |
|  | pr_CaPV-F15 | CTGTGAGGATTTCCAGCTT |
| 16 | pf_CaPV-F16 | CAAATTATATACACTTTAATGATGCTATG |
|  | pr_CaPV-F16 | CAAATCTTATTATATCTCCTTCTATTCC |
| 17 | pf_CaPV-F17 | TGGGGTAATTAGAAACGAGG |
|  | pr_CaPV-F17 | CACGAGTATGTTTTTACTGATGA |
| 18 | pf_CaPV-F18 | CCCAACATTATTTCCTTTTATGGT |
|  | pr_CaPV-F18 | CAATAAATGGATAAACTGAGGACA |
| 19 | pf_CaPV-F19 | CGTTTTCTAATTCTAACTTATTATAAACAG |
|  | pr_CaPV-F19 | GATACTGATATTCTTACAATGTCTATGA |
| 20 | pf_CaPV-F20V1 | GATCTCGTCCAGAAATATCAAC |
|  | pr_CaPV-F20V1 | GAACATCATGAAGAAGGAGAAG |
|  | pf_CaPV-F20V2 | AAAATCTCATAAAACTATTCTCAGACA |
|  | pr_CaPV-F20V2 | ATTTAAACATAGATTTTGGTTCAACTT |
| 21 | pf_CaPV-F21 | GTATTGATAAACAATACGCAATAACTAA |
|  | pr_CaPV-F21 | GCTATAATAGGTGAATACTCTGG |
| 22 | pf_CaPV-F22 | TTGAAAATGGACCAAAGATTAGGATA |
|  | pr_CaPV-F22 | CCATCTATTGATGATAATACAGAATAAG |
| 23 | pf_CaPV-F23 | CGATTACCTTCTCTTCTCGG |
|  | pr_CaPV-F23 | ATGAAATATATGGCAAAGTCATAG |
| 24 | pf_CaPV-F24 | CGTCTTCTATTATATGAAGTTTTCTG |
|  | pr_CaPV-F24 | CATCGTTGTCATGTTTGATATTG |
| 25 | pf_CaPV-F25 | GAGGAAAGCAAATGCTATGG |
|  | pr_CaPV-F25 | TCCTCAAATGCCATTGTCAC |
| 26 | pf_CaPV-F26 | TGTTGTGTTTTGGACGGTAC |
|  | pr_CaPV-F26 | CATAGACTCTTCTTTCGGTAGAC |
| 27 | pf_CaPV-F27 | GCAACATAGCAAAAGAAGAAGA |
|  | pr_CaPV-F27 | CCACTTTCTAACAACGTTGATAT |
| 28 | pf_CaPV-F28 | TGATATAAATCAAAAATCAGAAAGTGG |
|  | pr_CaPV-F28 | CTGTTGGTAATGTATTCCAGTAAG |
| 29 | pf_CaPV-F29 | TCGTTGAAAGTTATCAAGTATCTAATTTC |
|  | pr_CaPV-F29 | GTGCTACGTTATCCCACTTAAG |
| 30 | pf_CaPV-F30 | GAGATATACAAATTTTCACAATATTATGGTT |
|  | pr_CaPV-F30 | TTATTCAATAAGCCAATTAAACCTG |

pf: forward primer

pr: reverse primer
