# Supplementary material for: A robust, cost-effective and widely applicable whole-genome sequencing protocol for capripoxviruses
Source: J Virol Methods. 2022 Mar;301:114464. doi: 10.1016/j.jviromet.2022.114464 (PMC8872832; doi:10.1016/j.jviromet.2022.114464)
Supplement: Supplementary file 3 [file mmc3.docx]

Supplementary Table 3. Nucleotide modifications and their impact on the coding sequence of the attenuated LSDV strain SA-Neethling in comparison with the LSDV reference vaccine strain LW1959. Modifications confirmed by Sanger sequencing are highlighted in bold.

| Position on SA-Neethling | Nucleotide in SA-Neethling | Nucleotide in vaccine strain LW1959 (AF409138) | Nucleotide in virulent vaccine-associated Neethling genomes (MN636838-43) | Nucleotide in Neethling wild-type (NC_003027) | Modification located in homopolymer | ORF or IR^a^ affected | Impact on coding sequence |
| --- | --- | --- | --- | --- | --- | --- | --- |
| 538 | C | T | C | C | no | LW001 | N > D |
| 1480 | A | - | A | A | yes | IR |  |
| **2670** | **A** | **G** | **G** | **G** | **no** | **LW005** | **A > T** |
| **8309** | **T** | **-** | **-** | **-** | **yes** | **IR** |  |
| 10910 | - | T | - | T | yes | IR |  |
| **12210** | **T** | **-** | **-** | **-** | **yes** | **IR** |  |
| 13562 | A | - | A | A | no | LW019 | FRS^b^ |
| **13821** | **T** | **C** | **C** | **C** | **no** | **LW019** | **R > K** |
| **15484** | **A** | **-** | **-** | **-** | **no** | **LW022** | **Insertion DG** |
| **15484** | **C** | **-** | **-** | **-** | **no** | **LW022** |  |
| **15484** | **C** | **-** | **-** | **-** | **no** | **LW022** |  |
| **15484** | **A** | **-** | **-** | **-** | **no** | **LW022** |  |
| **15484** | **T** | **-** | **-** | **-** | **no** | **LW022** |  |
| **15484** | **C** | **-** | **-** | **-** | **no** | **LW022** |  |
| **16141** | **G** | **T** | **T** | **T** | **no** | **IR** |  |
| 16167 | A | - | A | - | yes | IR |  |
| **18893** | **-** | **T** | **T** | **T** | **yes** | **LW026** | **FRS** |
| 21735 | C | T | C | C | no | LW028 | T > A |
| 22783 | G | A | G | G | no | IR |  |
| 44014 | C | T | C | C | no | LW049 |  |
| 47178 | T | A | T | T | no | LW052 | N > K |
| 50336 | C | T | C | C | no | LW057 | I > V |
| **54455** | **C** | **T** | **T** | **T** | **no** | **LW062** |  |
| **68943** | **-** | **A** | **A** | **-** | **no** | **LW076** | **Deletion NND** |
| **68943** | **-** | **T** | **T** | **-** | **no** | **LW076** |  |
| **68943** | **-** | **A** | **A** | **-** | **no** | **LW076** |  |
| **68943** | **-** | **A** | **A** | **-** | **no** | **LW076** |  |
| **68943** | **-** | **T** | **T** | **-** | **no** | **LW076** |  |
| **68943** | **-** | **G** | **G** | **-** | **no** | **LW076** |  |
| **68943** | **-** | **A** | **A** | **-** | **no** | **LW076** |  |
| **68943** | **-** | **T** | **T** | **-** | **no** | **LW076** |  |
| **68943** | **-** | **A** | **A** | **-** | **no** | **LW076** |  |
| 77197 | T | A | T | T | no | LW083 | K > N |
| **77725** | **R** | **G** | **G** | **G** | **no** | **LW084** | **D > B** |
| 80648 | - | A | - | - | no | LW086 | FRS |
| 80705 | G | A | G | G | no | LW087 | G > D |
| 81256 | - | T | - | - | yes | LW087 | FRS |
| 81266 | C | T | C | C | no | LW087 | F > S |
| 81268 | A | T | A | A | no | LW087 | L > N |
| 81412 | A | - | A | A | no | LW087 | FRS |
| 81414 | A | - | A | A | no | LW087 | FRS |
| **83203** | **Y** | **T** | **T** | **T** | **no** | **LW088** | **K > R** |
| 91798 | G | A | G | G | no | LW098 | I > T |
| 92095 | T | C | T | T | no | LW098 | G > D |
| **99212** | **A** | **-** | **-** | **-** | **no** | **IR** |  |
| 105064 | G | T | G | G | no | LW112 | S > R |
| **106230** | **A** | **C** | **C** | **C** | **no** | **LW115** |  |
| 119587 | T | - | T | T | no | LW131a | FRS |
| 124401 | - | A | - | - | yes | LW134 | FRS |
| 129088 | T | C | T | T | no | LW135 |  |
| **136377** | **T** | **-** | **-** | **-** | **no** | **LW144** | **FRS** |
| **137122** | **T** | **G** | **G** | **G** | **no** | **LW144** |  |
| 150567 | G | A | G | G | no | LW156 | N > D |

^a^ IR: intergenic region

^b^ FRS: frameshift
