# Supplementary material for: A robust, cost-effective and widely applicable whole-genome sequencing protocol for capripoxviruses
Source: J Virol Methods. 2022 Mar;301:114464. doi: 10.1016/j.jviromet.2022.114464 (PMC8872832; doi:10.1016/j.jviromet.2022.114464)
Supplement: Supplementary file 4 [file mmc4.docx]

SUPPLEMENTARY DATA


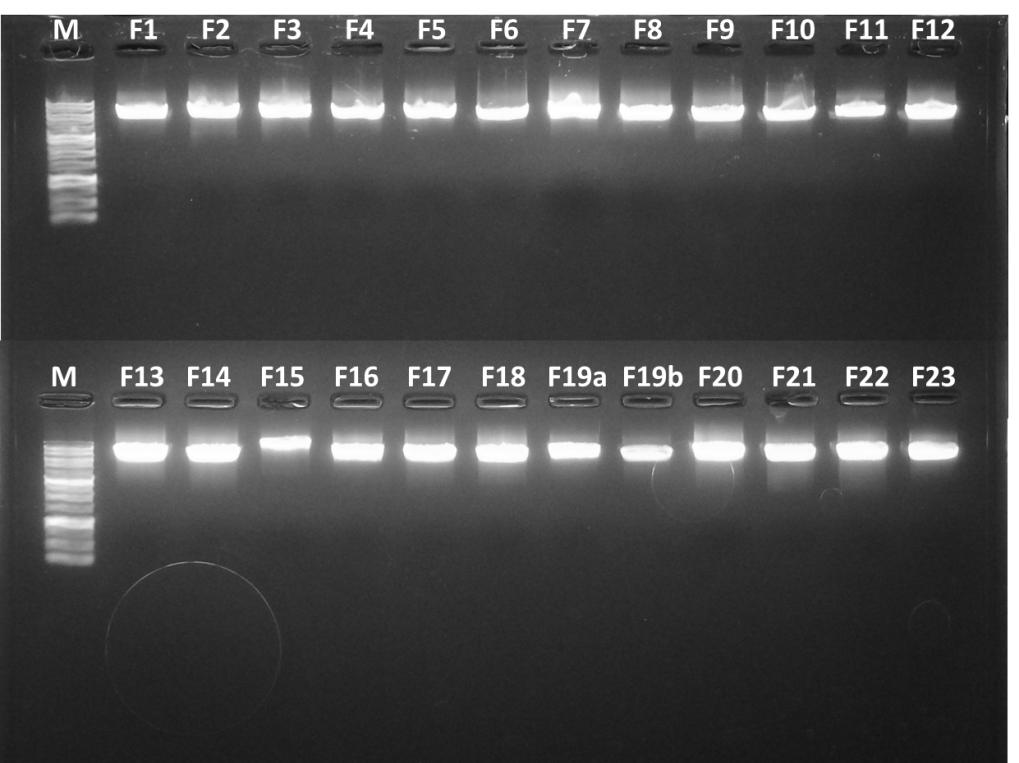


Supplementary Fig1. 1% agarose gel electrophoreses analysis of the 23 long range PCR amplicons for the GTPV strain Gorgan from Caprivac vaccine (Jovac) . M= molecular weight marker range 250 – 10,000 bp.


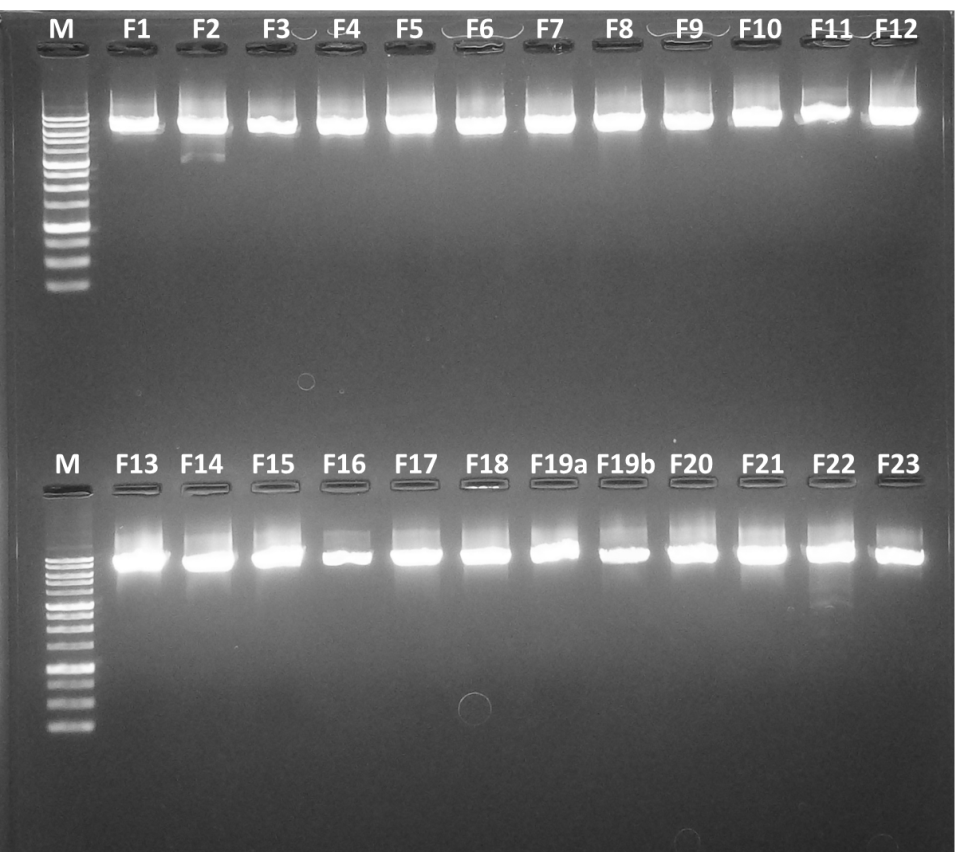


Supplementary Fig2. 1% agarose gel electrophoreses analysis of the 23 long range PCR amplicons for the Yugoslavian SPPV strain RM65 from Jovivac vaccine (Jovac). M= molecular weight marker range 250 – 10,000 bp.


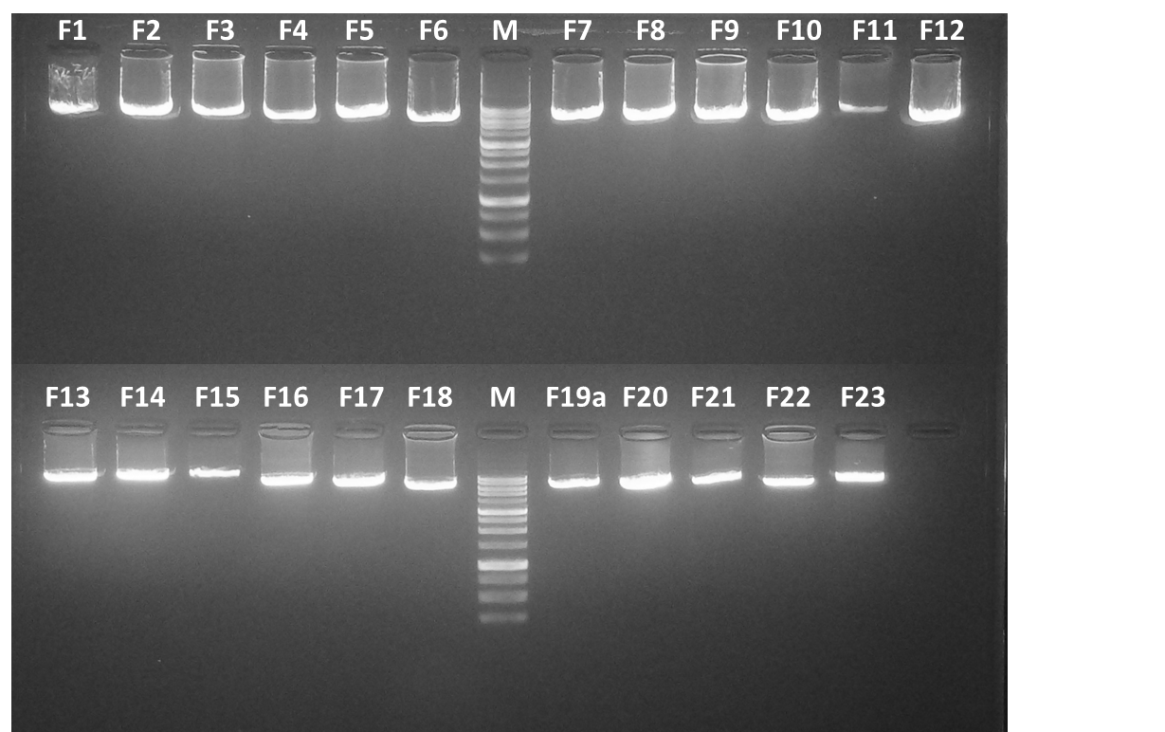


Supplementary Fig3. 1% agarose gel electrophoreses analysis of the 23 long range PCR amplicons for the attenuated LSDV strain SA-Neethling skin lesion sample. M= molecular weight marker range 250 – 10,000 bp.
